# Prognostic Effect of Inflammatory Genes on Stage I–III Colorectal Cancer—Integrative Analysis of TCGA Data

**DOI:** 10.3390/cancers13040751

**Published:** 2021-02-11

**Authors:** Eun Kyung Choe, Sangwoo Lee, So Yeon Kim, Manu Shivakumar, Kyu Joo Park, Young Jun Chai, Dokyoon Kim

**Affiliations:** 1 Department of Surgery, Seoul National University Hospital Healthcare System Gangnam Center, Seoul 06236, Korea; choe523@snu.ac.kr; 2 Department of Biostatistics, Epidemiology & Informatics, Perelman School of Medicine, University of Pennsylvania, Philadelphia, PA 19104-6116, USA; jebi1771@ajou.ac.kr (S.Y.K.); Manu.Shivakumar@pennmedicine.upenn.edu (M.S.); 3 Department of Surgery, Seoul National University College of Medicine, Seoul 03080, Korea; kjparkmd@snu.ac.kr; 4 Department of Future Convergence, Cyber University of Korea, Seoul 03051, Korea; sangwoo07lee@cuk.edu; 5 Department of Software and Computer Engineering, Ajou University, Suwon 16499, Korea; 6 Department of Surgery, Seoul Metropolitan Government—Seoul National University Boramae Medical Center, Seoul 07061, Korea; kevinjoon@snu.ac.kr; 7 Institute for Biomedical Informatics, University of Pennsylvania, Philadelphia, PA 19104-6116, USA

**Keywords:** colorectal cancer, inflammatory genes, multi-omics data, TCGA

## Abstract

**Simple Summary:**

Research interest in the role of inflammation in the progression and prognosis of colorectal cancer (CRC) is growing. In this study, we evaluated the expression and DNA methylation levels of inflammation-related genes in CRC tissues using the TCGA-COREAD dataset by integratively combining multi-omics features using machine learning. Statistical analysis was additionally performed to allow for interpretable, understandable, and clinically practical results. An integrative model combining expression, methylation, and clinical features had the highest performance. In multivariate analysis, the methylation levels of CEP250, RAB21, and TNPO3 were significantly associated with overall survival. Our study results implicate the importance of integrating expression and methylation information along with clinical information in the prediction of survival. CEP250, RAB21, and TNPO3 in the prediction model might have a crucial role in CRC prognosis and further improve our understanding of potential mechanisms linking inflammatory reactions and CRC progression.

**Abstract:**

Background inflammatory status indicators have been reported as prognostic biomarkers of colorectal cancer (CRC). However, since inflammatory interactions with the colon involve various modes of action, the biological mechanism linking inflammation and CRC prognosis has not been fully elucidated. We comprehensively evaluated the predictive roles of the expression and methylation levels of inflammation-related genes for CRC prognosis and their pathophysiological associations. Method. An integrative analysis of 247 patients with stage I-III CRC from The Cancer Genome Atlas was conducted. Lasso-penalized Cox proportional hazards regression (Lasso-Cox) and statistical Cox proportional hazard regression (CPH) were used for the analysis. Results. Models to predict overall survival were designed with respective combinations of clinical variables, including age, sex, stage, gene expression, and methylation. An integrative model combining expression, methylation, and clinical features performed better (median C-index = 0.756) than the model with clinical features alone (median C-index = 0.726). Based on multivariate CPH with features from the best model, the methylation levels of CEP250, RAB21, and TNPO3 were significantly associated with overall survival. They did not share any biological process in functional networks. The 5-year survival rate was 29.8% in the low methylation group of CEP250 and 79.1% in the high methylation group (*p* < 0.001). Conclusion. Our study results implicate the importance of integrating expression and methylation information along with clinical information in the prediction of survival. CEP250, RAB21, and TNPO3 in the prediction model might have a crucial role in CRC prognosis and further improve our understanding of potential mechanisms linking inflammatory reactions and CRC progression.

## 1. Introduction

Oncologic research on the role of inflammation in the progression and prognosis of cancer is growing [1]. Candidate inflammatory biomarkers, such as neutrophils, lymphocytes, monocytes, platelets, the lymphocyte-to-monocyte ratio (LMR), the neutrophil-to-lymphocyte ratio (NLR), and the platelet-to-lymphocyte ratio (PLR), have been suggested as predictive factors for cancer prognosis [2,3,4]. In the colorectum, molecular pathophysiological analyses imply that inflammation promotes the progression of colorectal cancer [5]. The role of inflammation in the colorectum is well manifested in inflammatory bowel diseases such as ulcerative colitis and Crohn’s disease. In these patients, chronic inflammation is a major risk factor for the development of gastrointestinal malignancies, especially colorectal cancer (CRC) [6]. Inflammatory biomarkers such as NLR, LMR, and PLR have been suggested as prognostic markers in colorectal cancer [7,8,9,10]. Chronic inflammation can trigger genetic mutations and epigenetic alterations that promote malignant cell transformation [11]. However, inflammatory interactions with the colon involve various modes of action, such as immune cells, cytokines, and other immune mediators, in virtually all sequences of CRC progression, including initiation, progression, and metastasis [12]. In recent reports, it has been postulated that epigenetic changes have a critical role in establishing efficient expression profiles during inflammation and disease [13,14,15]. Due to the complexity of the association between inflammation and CRC, the underlying mechanism linking inflammation and CRC prognosis has not been fully elucidated. To elucidate this mechanism, integrative analysis of multi-omics data with a genome-wide view is necessary [16].

The Cancer Genome Atlas (TCGA) has a collection of genomic and epigenomic data along with the clinical data of patients for a large number of cancer types [17]. The TCGA aims to develop a catalog of major cancer genomics through integrated multidimensional analyses [18]. This goal has increasingly fueled the prediction of various oncological outcomes based on multi-omics data [19,20,21,22].

In this study, we systematically evaluated the expression and DNA methylation levels of inflammation-related genes in CRC tissues using the TCGA-COREAD dataset. We comprehensively analyzed the whole set of inflammation-related genes based on both RNA expression and methylation to evaluate the predictive roles of these genes in overall survival and examine their significance levels in association with overall survival. By using Lasso-penalized Cox proportional hazards regression (Lasso-Cox), features were selected to design the prediction model for overall survival and used to evaluate whether the genetic features could improve the prediction performance in addition to clinical prognostic factors such as age and TNM staging. To our knowledge, no previous studies have focused on the use of integrated omics biomarkers, particularly inflammation-related genes, as additive features to the basis of the current TNM staging system in designing a prediction model for overall survival in colorectal cancer.

## 2. Methods

### 2.1. Data Acquisition and Preprocessing

We utilized the clinical information, RNA-seq gene data, and DNA methylation profiles of The Cancer Genome Atlas-colorectal adenocarcinoma (TCGA-COREAD). Clinical information and RNA-seq gene expression were downloaded from the UCSC Xena browser (https://xenabrowser.net (accessed on 4 May 2019)). Gene-level methylation data of TCGA-COREAD were retrieved from the Broad Institute Firebrowse (http://firebrowse.org (accessed on 4 May 2019)). The gene expression data were acquired using Illumina HiSeq 2000 RNA sequencing. The gene expression transcription estimates are shown as log-transformed RSEM normalized counts. For the RNA-seq data, genes that had more than 50% samples with 0 expression levels were removed. For the methylation data, we used the methylation data by picking the probe with the minimum correlation with mRNA-seq data for each gene. Genes that had zero methylation levels in all samples were excluded. Two authors (EKC, KJP) who are colorectal surgeons independently reviewed the pathological report and revised incorrect or inconsistent clinical information. Among the 736 samples in the data, we excluded the samples based on the clinical information as follows: samples collected from normal tissue; stage IV colorectal cancer; missing data on pathological stages, survival data, and age information; and follow-up duration less than 12 months. For clinical information, age, sex, TNM stage, overall survival, and event status were used as clinical variables. Finally, we chose the data for analysis using the overlapping sample IDs among the RNA-seq, methylation and clinical data sets. Thus, 247 patients were used for further analyses.

### 2.2. Ethics Statement

On TCGA publication guidelines (http://cancergenome.nih.gov/publications/publicationguidelines (accessed on 4 May 2019)), there are no restrictions on the publication or use of the dataset. The study was conducted in accordance with the Declaration of Helsinki.

### 2.3. Feature Selection

**Figure 1:**
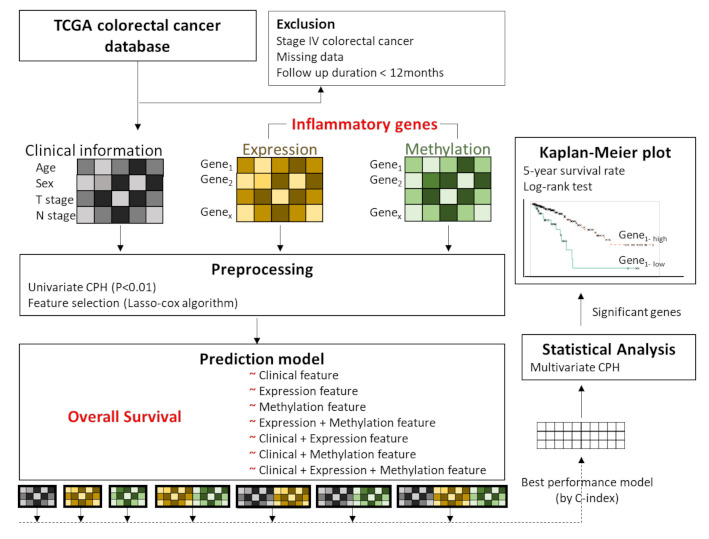
Overview of the analysis framework. We preprocessed the gene sets related to inflammatory status and clinical information, based on the significance of association with overall survival by performing univariate Cox proportional hazard regression (CPH) analysis. These features passed the suggestive significance level (*p* < 0.01). Features with concatenated expression and methylation were also included. Feature selection was performed by using Lasso-penalized Cox proportional hazards regression (Lasso-Cox). Seven modes were designed by respective combinations of features, and the performances were evaluated by Lasso-Cox according to the C-index. Among the models, the highest C-index model was used for multivariate CPH analysis of overall survival. Then, Kaplan–Meier analysis was performed for the genes that were significantly associated with overall survival, and the log rank test was performed for comparisons of each group.

Figure 1 shows an overview of the analysis framework. First, we selected the gene sets related to the inflammatory status from the National Center for Biotechnology Information’s (NCBI) gene database (www.ncbi.nlm.nih.gov/gene (accessed on 15 May 2019)) [23], with the filtering term “(inflammatory) AND “homo sapiens”[porgn: _txid9606]”. A total of 2576 genes were retrieved and used as inflammation-related genes for the analyses. Then, we preprocessed the gene sets and the clinical information (such as age (<65 years vs. ≥65 years), sex (male vs. female), T stage (T1, T2 vs. T3, T4), and N stage (N0 vs. N1, N2) based on the significance of their association with overall survival determined by univariate Cox proportional hazard regression (CPH) analysis. The clinical, expression, and methylation features that passed the suggestive significance level (*p* < 0.01) were used for further analyses (Clinical, Expression, Methylation). We also made an input matrix in which expression and methylation data were concatenated (Expression + Methylation) (Figure 1). Finally, feature selection was performed for the respective data sets (Clinical, Expression, Methylation, and Expression + Methylation) by using Lasso-penalized Cox proportional hazards regression (Lasso-Cox). The training set versus test set ratio was 8:2. We selected important features that showed the best prediction performance by training the Lasso-Cox model in the Clinical, Expression, Methylation, and Expression + Methylation dataset. To validate the feature selection model, we performed 50 iterations of 5-fold cross-validation in the training set.

### 2.4. Overall Survival Prediction Based on Omic Features

Using the selected features, we trained prediction models for overall survival using Lasso-penalized Cox proportional hazards regression and compared the performances of each model. Seven models were designed by combining features such as clinical features (C); Expression features (E); Methylation features (M); Expression + Methylation features (EM); Clinical features + Expression features (C-E); Clinical features + Methylation features (C-M); and Clinical features + Expression + Methylation features (C-EM). The performances among the models were compared by the concordance index (C-index), which measures how concordant the observations and predictions by our model are for a pair of randomly chosen patient samples [24] (Figure 1). After performing the analysis using machine learning, we tried to apply statistical analysis to the model to make the results interpretable and evident for clinical utility. Among the models, we used the model that achieved the highest C-index to perform multivariate CPH analysis to examine which features had a significant association with overall survival (*p* < 0.05). Then, Kaplan–Meier analysis was carried out using the genes that were significantly associated with overall survival. The optimal cutoff points for those genes, to divide patients into two groups, were determined by MaxStat packages in R (maximally selected rank statistics). MaxStat performs a test of independence of response and one or more covariables using maximally selected rank statistics [25]. The log-rank test was used to compare the survival curves of the different gene-level groups. Kaplan–Meier plots were visualized by the “ggkm” R package. Finally, integrated multispecies prediction (IMP) [26] was used to visualize the gene-gene network and biological process network. IMP integrates multiple sources of evidence for functional interactions by integrating 3741 genome-scale datasets for 7 organisms and predicts 582 disease and 12,117 biological processes (http://imp.princeton.edu/ (accessed on 29 July 2019)) [27]. We queried IMP with the gene set from the best prediction model. All statistical and computational analyses were performed using R statistical software (version 3.5.3 R development Core Team; R Foundation for Statistical Computing, Vienna, Austria). The putative association between clinical information and survival outcome was assessed by Chi-square test, Student’s t-test and analysis of variance (ANOVA) for independent groups in Table 1.

## 3. Results

### 3.1. Patient Demographics

Our study population comprised 247 patients (131 males and 116 females) who underwent colectomy for stage I–III colorectal cancer. The mean patient age was 64.28 ± 12.90 years, and the median follow-up was 26.83 months (ranging from 12.17 to 150.07 months). Patients’ characteristics are shown in Table 1.

### 3.2. Feature Selection for Overall Survival with Lasso-Cox

We preprocessed the gene sets and the clinical information based on the significance of association with overall survival by performing univariate CPH analysis. Features that passed the suggestive significance level (*p* < 0.01) were 4 clinical variables (age, sex, T stage, and N stage), 26 gene expression and 14 methylation features. By concatenating the selected expression and methylation features, Expression + Methylation data had 40 features. After feature selection using Lasso-Cox, 4 variables were selected for clinical features (C), 16 for expression features (E), 12 for methylation features (M), and 14 for omic features from the combined dataset (EM). The list of the features is shown in Table S1.

### 3.3. Training Prediction Models for Overall Survival with Lasso-Cox

Using various combinations from 4 feature sets (C, E, M, and EM), we designed seven models to predict overall survival (Figure 1). The performances of each model are shown in Table 2. Among the 4 respective feature sets, a model with EM showed the highest performance (median C-index = 0.727). In particular, among models incorporating the genetic features into clinical features, an integrative model with C-EM showed the best performance (median C-index = 0.756) (Table 2). Adding the respective E or M to C did not improve the performance compared to the model with C alone; however, integrating the EM and C showed great improvement in our prediction model.

### 3.4. Association Analysis of Overall Survival with Multivariate Cox Proportional Hazard Regression

**Figure 2:**
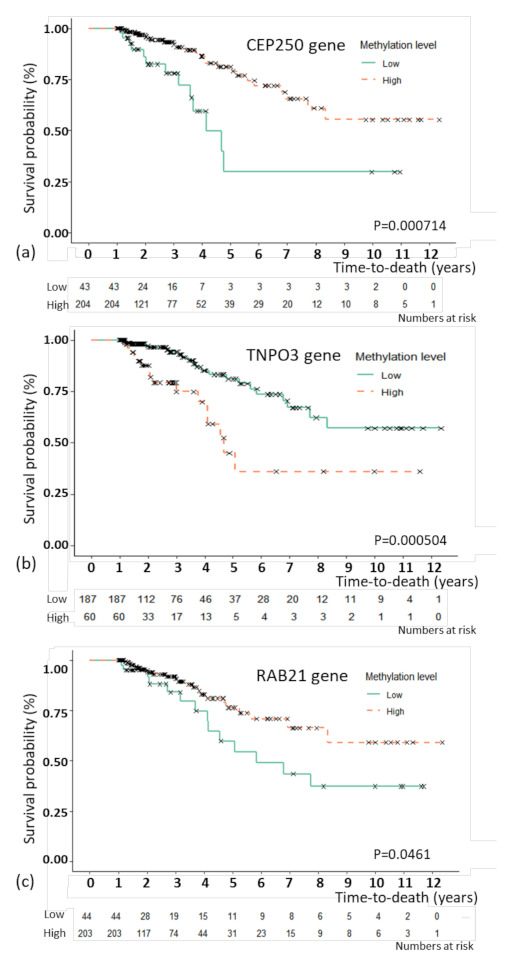
Kaplan–Meier plot of overall survival with genes that were significant in the multivariate Cox proportional hazard regression analysis. Kaplan–Meier plot of overall survival with the CEP250 (**a**), TNPO3 (**b**), and RAB21 (**c**) genes. The patients were divided by the optimal cutoffs for each feature based on MaxStat (−0.923 for CEP250; 0.5 for TNPO3, and −0.367 for RAB21).

For the model with C-EM, the best prediction model, an association study was performed to examine the association between the selected features and overall survival. By multivariate CPH, CEP250 (*p* = 0.035), RAB21 (*p* = 0.002), and TNPO3 (*p* = 0.011) as methylation features showed independent associations with overall survival (Table 3). For the three methylation features, Kaplan–Meier plots were generated (Figure 2). The patients were divided by the optimal cutoffs for each feature based on MaxStat (−0.923 for CEP250; 0.5 for TNPO3, and −0.367 for RAB21). Figure 2 shows the Kaplan–Meier plots with the results of the log-rank test. The 5-year survival rate of TNPO3 was 81.1% for methylation below the cutoff point and 76.5% for methylation above the cutoff point; for RAB21, the rate was 54.4% for methylation below the cutoff point and 76.5% for methylation above the cutoff point; and for CEP250, the rate was 29.8% for methylation below the cutoff point and 79.1% for methylation above the cutoff point.

### 3.5. Gene-Gene Network and Biological Process Network

**Figure 3:**
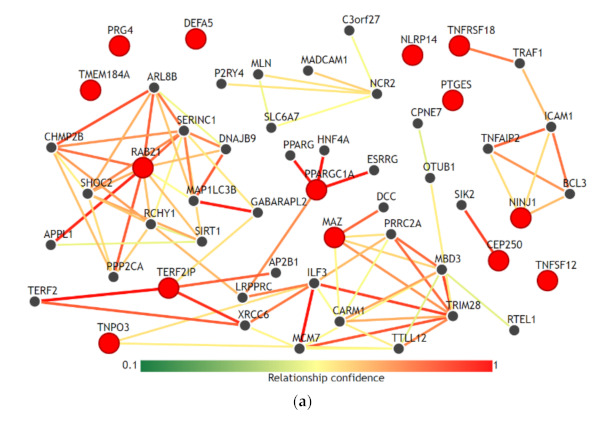

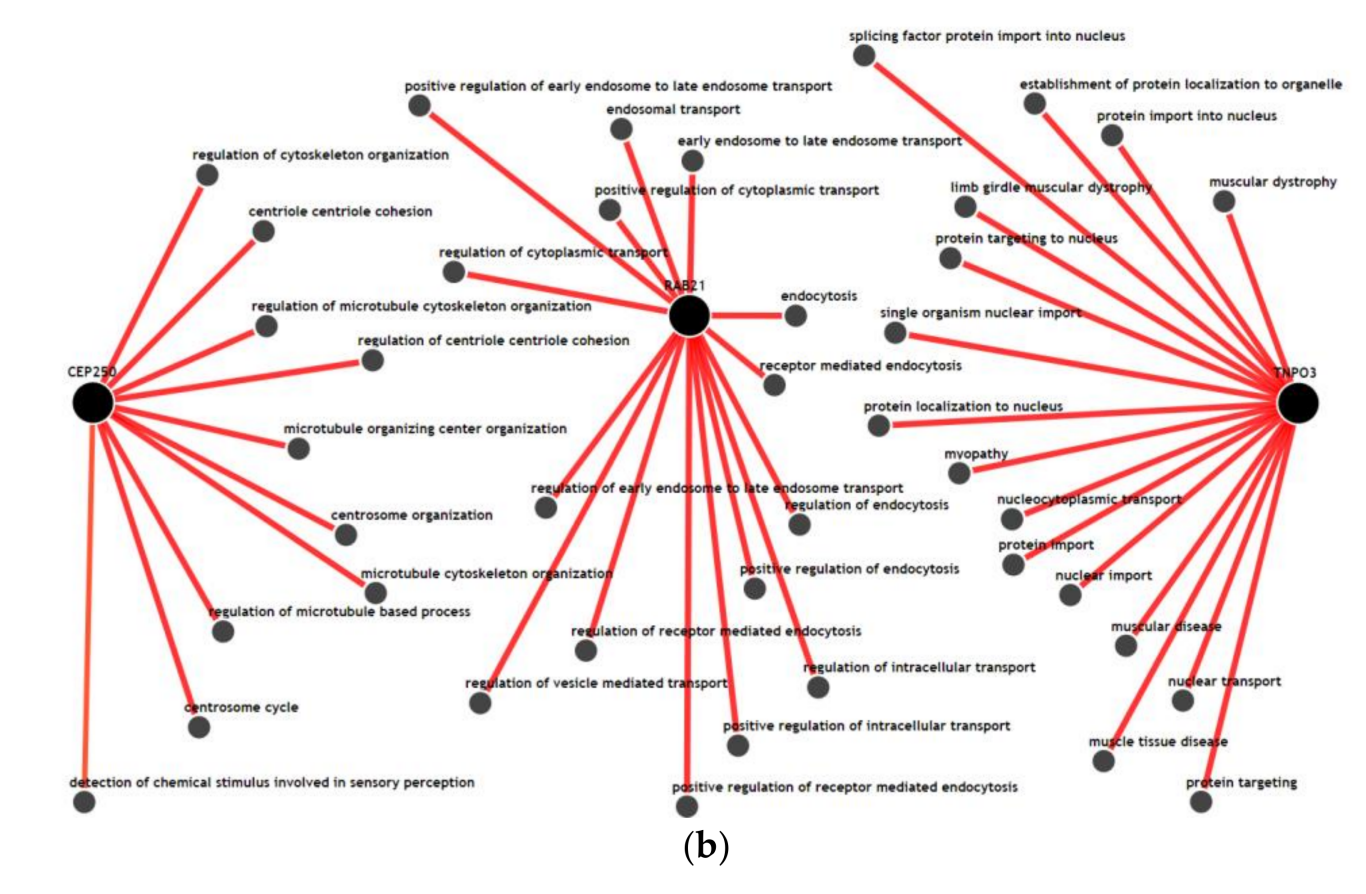
Functional network of the gene features used in the integrative model with clinical, gene expression and methylation features for the prediction of overall survival. (**a**) Gene-gene networks queried by 14 genes in the model. Red dots are the queried genes (14 genes, Table S1), and the black dots are the genes predicted based on functional networking analysis (0.5 confidence with 40 genes). Except for RAB21, TERF21P, and PPARGC1A, most of the genes among the 14 genes had fewer than 3 strong relationships with other genes by network. (**b**) Biological process networks queried by 3 genes (CEP250, TNPO3, and RAB21) that showed a significant association with overall survival (confidence 0.5). All genes had unique biological processes, and there were no shared biological processes among the three genes.

We visualized the functional network of the expression and methylation features used in the prediction model C-EM using IMP. For gene-gene networking construction, we queried all 14 genes used in the model, and the results are shown in Figure 3a. Except for RAB21, TERF21P, and PPARGC1A, 11 genes among 14 genes had less than 3 strong relationships with other genes in the network. The biological process network for the 3 genes, which are significantly associated with overall survival, was also visualized (Figure 3b). We used a confidence level of 0.5 when visualizing networking. Notably, all genes had unique biological processes, and there were no shared biological processes among the three genes.

## 4. Discussion

Age, sex, T stage, and N stage, which are used in this study as clinical features, are the most commonly used clinical factors for predicting the prognosis and survival of patients with colorectal cancer [28]. Comparing the prediction performances of these clinical factors, the integrative model that integrated clinical, expression and methylation features significantly improved the performance of predicting overall survival. Adding expression features alone or methylation features alone to the clinical features did not contribute to the improvement in performance. However, only the combined dataset between expression and methylation features was able to improve the performance. This finding might reflect the fact that inflammatory interactions with CRC include a wide range of biological processes in immune cells, cytokines, and other immune mediators in virtually all sequences of CRC progression, including initiation, progression, and metastasis [12]. This result is also consistent with our results using IMP. In the gene-gene network (Figure 3a), the selected genes in the prediction model did not have high-dimensional networks among each other, except for RAB21 and PPARGC1A, and all genes had fewer or no connections to other genes. This could imply that these genes uniquely contribute to the inflammation-cancer progression mechanism. In addition, in the biological process network (Figure 3b), the genes associated significantly with overall survival (RAB21, CEP250, and TNPO3) did not share any biological processes. This seems to be in accordance with the finding that they were statistically independent factors for association with overall survival.

In the best integrative prediction model, 7 expression and 7 methylation features were selected. We performed a literature review on the function of these gene features and their relationship with inflammation. The CEP250 gene and TERF2IP gene are involved in the cell cycle. The CEP250 gene encodes a centrosomal protein contributing to centrosome–centrosome cohesion during interphase of the cell cycle [29]. It has been reported to be associated with inflammation in joints and bones [30]. There is also a genome-wide association study that shows the association of CEP250 with inflammatory bowel disease [31]. TERF21P encodes a protein involved in telomere length regulation [29]. Several studies have shown that their aberrant activation leads to increased cancer cell proliferation and tumorigenesis [32,33].

The RAB21 gene and NINJ1 gene are related to cell adhesion and migration. The RAB21 gene is a Rab family of monomeric GTPases, and the encoded protein is related to the regulation of cell adhesion and migration [23]. It is involved in the inflammatory reaction by regulating lipopolysaccharide-induced proinflammatory responses [34]. It has been reported to be related to cancer invasion and cancer mobility [35,36]. The NINJ1 gene produces a protein that is a homophilic cell adhesion molecule [37] and plays a role in the progression of multiple sclerosis [38]. NINJ1 is a target of p53 and has been known to repress WT p53 expression [39].

The TNPO3, MAZ, and PPARGC1A genes are related to gene regulation. The TNPO3 gene encodes a nuclear import receptor for serine/arginine-rich (SR) proteins [29]. This gene is reported to be associated with several inflammatory autoimmune diseases, such as systemic lupus erythematosus [40] and rheumatoid arthritis [41]. There are no reports related to colon disease or cancer. The MAZ gene affects the expression of MYC gene [42]. The MYC gene is well known for its role in the progression of CRC [43]. The MYC gene is frequently deregulated in inflammation, and its expression is affected by DNA methylation [11]. The PPARGC1A gene encodes a transcriptional coactivator that regulates the genes involved in energy metabolism [29]. In an experiment, it was profoundly reduced in ulcerative colitis patients [44].

There are several genes related to cytokine reactions. The TNFRSF18 and TNFSF12 genes are related to tumor necrosis factor (TNF), which is a well-known inflammatory biomarker that promotes cytokines in colon cancer [12]. The NLRP14 gene encodes the NLRP (nucleotide-binding oligomerization domain, leucine-rich repeat and pyrin domain containing) protein family, which belongs to the inflammasome and activates proinflammatory enzymes [29]. The NLRP inflammasome is suggested as a possible linkage mechanism between obesity-associated low-grade chronic inflammation and CRC development [45]. The PTGES gene encodes a glutathione-dependent prostaglandin E synthase, and its expression seems to be induced by the proinflammatory cytokine interleukin 1 beta (IL1B) [29]. PTGES is upregulated in CRC and premalignant lesions such as colonic adenomatous polyps [46,47]. The DEFA5 gene encodes a family of antimicrobial and cytotoxic peptides thought to be involved in host defense [29]. It is reported as a candidate biomarker for Crohn’s disease [48]. The PRG4 gene encodes a large proteoglycan [29]. It plays an important anti-inflammatory role in osteoarthritis synoviocyte proliferation [49]. In addition, PRG4 has been demonstrated to have anti-inflammatory properties [50], and it may suppress breast cancer cell invasion [51]. There are no reports related to colon disease or cancer.

For the TMEM184A gene, there are few reports on its pathophysiology in inflammation and cancer progression. A few reports have shown that TMEM 184A functions as a heparin receptor and regulates the anti-inflammatory response of endothelial cells [52].

As seen in the literature review, the selected inflammatory genes have a variety of pathophysiological mechanisms that could be considered to contribute to inflammation-CRC linkage mechanisms. These genes could be the crucial sets of genes that could cover the majority of the mechanism linking inflammation—CRC progression. In addition, it could be postulated that the role of inflammatory genes in CRC progression is not related or connected to one functional pathway but is affected through multiple global mechanisms by respective inflammatory genes.

This study has several advantages. We comprehensively analyzed all genes related to inflammation to see the global network between inflammation and CRC progression and included both expression and methylation data for those genes. By combining the expression and methylation data in the model design, we were able to obtain a survival prediction model that improved the performance of conventional clinical prediction models. This performance would not be achievable if the model were to be designed using either gene expression or methylation data. Second, we performed both the prediction model based on computational analysis and the association study based on statistical analysis. This will help to interpret the results more intuitively and support evidence for the results of the prediction model.

However, there are a couple of limitations in our analysis. First, as we used an open-source database, the samples were not collected under well-controlled and unified conditions. The resulting heterogeneity of the samples could have made the characteristics of the population different from the real world population of CRC patients. We compared the 5-year overall survival rate in respective pathologic stages from our study population to that of colorectal cancer patients from the Surveillance, Epidemiology, and End Results (SEER) program, which is a large-scale data set from the United States [53]. The patients from our TCGA data set showed that patients with stage I, II, and III colorectal cancer had 5-year overall survival rate of 83.1%, 80.3%, and 55.0%, respectively, as compared with 82.7%, 70.3%, and 68.7% for those from the SEER program [54]. The 5-year overall survival rate in stage I was similar between two data sets. In stages II and III, which the prognosis is highly affected by various clinical factors other than overall stage, there were notable differences in the 5-year overall survival rates between two data sets. In the TCGA data set, which is an open-source database, there were many missing data. This prohibited us from using important clinical factors such as adjuvant therapy protocol, microsatellite instability, lymphatic invasion, and venous invasion for survival analysis. These clinical factors could also have a crucial role in predicting overall survival. The limitation of information on the study population characteristics might have limited the extent to which our study reflects the findings in the real world population with colorectal cancer. Second, we simply concatenated the clinical factors, RNA expression and methylation levels for integration. Transformational integration could be applied in a larger set of samples. Third, since there are no open-source databases that include both RNA expression and methylation data with long-term survival information, we could not validate the results in another data set. This should be performed in future studies by constructing a colorectal cancer cohort with multi-omics profiles and long-term outcome information.

## 5. Conclusions

By analyzing colorectal cancer patients from TCGA datasets, we were able to develop prediction models for overall survival based on genetic profiles in relation to the inflammatory status. Overall survival was best predicted by the model combining expression and methylation features with clinical features, which outperformed the model designed using clinical features alone. This emphasizes the importance of introducing integrative omics biomarkers of the inflammation status as additive features to the current TNM staging system to improve prognostic predictions in patients with colorectal cancer. Functionally, the gene features of this model rarely share a functional network, implying that the inflammatory status contributes to colorectal cancer progression, not in a simple biological process but rather a global gene set linkage of biological processes. By association analysis, we were able to identify that the methylation levels of the CEP250, RAB21, and TNPO3 genes independently play crucial roles in colorectal cancer prognosis. These findings could be used to elucidate the underlying mechanism between the inflammatory status and colorectal cancer progression. We believe that the collection of various types of omics data from colorectal cancer patients in future studies will enable a better understanding of the pathophysiology of its disease mechanism.

## Figures and Tables

**Table 1:** Demographic features of the study population.

Features	Alive	Dead	*p* Value
Age	62.9 ± 12.5	71.7 ± 12.6	<0.001
<65	111 (53.1%)	7 (18.4%)	
≥65	98 (46.9%)	31 (81.6%)	
Continuous	62.9 ± 12.5	71.7 ± 12.6	
Gender			0.407
Male	108 (51.7%)	23 (60.5%)	
Female	101 (48.3%)	15 (39.5%)	
Overall stage			0.201
Stage 1, 2	136 (65.1%)	20 (52.6%)	
Stage 3	73 (34.9%)	18 (47.4%)	
Stage 1	46 (22.0%)	2 (5.3%)	
Stage 2	90 (43.1%)	18 (47.4%)	
Stage 3	73 (34.9%)	18 (47.4%)	
T stage			0.046
T1, T2	50 (23.9%)	3 (7.9%)	
T3, T4	159 (76.1%)	35 (92.1%)	
T1	8 (3.8%)	1 (2.6%)	
T2	42 (20.1%)	2 (5.3%)	
T3	146 (69.9%)	32 (84.2%)	
T4	13 (6.2%)	3 (7.9%)	
N stage			0.113
LN negative	136 (65.1%)	19 (50.0%)	
LN positive	73 (34.9%)	19 (50.0%)	
N0	136 (65.1%)	19 (50.0%)	
N1	49 (23.4%)	9 (23.7%)	
N2	24 (11.5%)	10 (26.3%)	
Tumor location			1
Right colon	102 (50.2%)	17 (48.6%)	
Left colon	101 (49.8%)	18 (51.4%)	
Venous invasion			0.916
Negative	147 (80.8%)	25 (78.1%)	
Positive	35 (19.2%)	7 (21.9%)	
Lymphatic invasion			0.887
Negative	140 (75.7%)	24 (72.7%)	
Positive	45 (24.3%)	9 (27.3%)	
Follow up duration (months)	38.54 ± 31.26	39.97 ± 22.79	0.739

**Table 2:** The prediction performances of each model were compared by the concordance index.

Features Used	Min.	1st Qu.	Median	Mean	3rd Qu.	Max.
Clinical features	0.318	0.648	0.726	0.706	0.791	0.921
Expression features	0.449	0.614	0.691	0.688	0.774	0.899
Methylation features	0.447	0.580	0.686	0.683	0.772	0.899
Expression features + Methylation features	0.337	0.647	0.727	0.715	0.826	0.884
Clinical features + Expression features	0.438	0.609	0.667	0.673	0.761	0.832
Clinical features + Methylation features	0.333	0.628	0.704	0.682	0.757	0.866
Clinical features + Expression features + Methylation features	0.326	0.655	0.756	0.708	0.818	0.883

**Table 3:** Association analysis of the integrative model with clinical, gene expression and methylation features and overall survival. Analysis was performed with multivariate Cox proportional hazard regression. CEP250, centrosomal protein 250; DEFA5, defensin alpha 5; MAZ, MYC-associated zinc finger protein; NINJ, nerve injury-induced protein 1; NLRP14 (expression), NLR family pyrin domain-containing 14; PPARGC1A, peroxisome proliferator-activated receptor γ coactivator 1 alpha; PRG4, proteoglycan 4; PTGES, prostaglandin E synthase; RAB21, member of the RAS oncogene family; TERF2IP, TERF2-interacting protein; TMEM184A, transmembrane protein 184A; TNFRSF18, TNF receptor superfamily member 18; TNFSF12 TNF superfamily member 12; TNPO3, transportin 3.

Features	Hazard Ratio (HR)	95% CI, Lower	95% CI, Upper	Z Value	Adjusted *p* Value
Age	1.697	0.609	4.733	1.011	0.312
N stage	2.942	1.253	6.912	2.477	0.013
T stage	0.919	0.237	3.557	−0.122	0.903
Gender	0.910	0.390	2.121	−0.219	0.827
CEP250 (methylation)	0.592	0.364	0.963	−2.110	0.035
DEFA5 (expression)	0.786	0.462	1.337	−0.888	0.374
MAZ (methylation)	0.967	0.809	1.156	−0.369	0.712
NINJ1 (methylation)	1.339	0.910	1.968	1.482	0.138
NLRP14 (expression)	0.797	0.525	1.211	−1.063	0.288
PPARGC1A (expression)	0.808	0.636	1.027	−1.744	0.081
PRG4 (expression)	1.256	0.871	1.811	1.219	0.223
PTGES (expression)	1.399	0.937	2.087	1.644	0.100
RAB21 (methylation)	1.556	1.172	2.065	3.060	0.002
TERF2IP (expression)	1.452	0.893	2.360	1.502	0.133
TMEM184A (expression)	1.251	0.819	1.911	1.038	0.299
TNFRSF18 (methylation)	1.489	0.783	2.829	1.214	0.225
TNFSF12 (methylation)	1.132	0.801	1.600	0.704	0.481
TNPO3 (methylation)	1.465	1.092	1.967	2.543	0.011

## Data Availability

Clinical and RNA-seq data from The Cancer Genome Atlas-colorectal adenocarcinoma (TCGA-COREAD) are available from the UCSC Xena browser (https://xenabrowser.net). Gene-level methylation data from TCGA-COREAD are available from the Broad Institute Firebrowse (http://firebrowse.org (accessed on 4 May 2019)).

## Supplementary Materials

The following are available online at https://www.mdpi.com/2072-6694/13/4/751/s1, Table S1. Selected features by Lasso-Cox feature selection.

## Abbreviations

CRC	Colorectal
TCGA-COREAD	The Cancer Genome Atlas-Colorectal Cancer
Lasso-Cox	Lasso-penalized Cox proportional hazards regression
CPH	Cox proportional hazard regression
IMP	Integrative multispecies prediction
LMR	Lymphocyte-to-monocyte ratio
NLR	Neutrophil-to-lymphocyte ratio
PLR	Platelet-to-lymphocyte ratio
C	Clinical features
E	Gene expression features
M	Gene methylation features
EM	Concatenated expression and methylation features
