# Supplementary material for: Prognostic Effect of Inflammatory Genes on Stage I–III Colorectal Cancer—Integrative Analysis of TCGA Data
Source: Cancers (Basel). 2021 Feb 11;13(4):751. doi: 10.3390/cancers13040751 (PMC7916934; doi:10.3390/cancers13040751)
Supplement: Supplementary file 1 [file cancers-13-00751-s001.pdf]

cancers

MDPI

Supplementary Material

Prognostic Effect of Inflammatory Genes on Stage I-III Colorectal Cancer—Integrative Analysis of TCGA Data

Eun Kyung Choe, Sangwoo Lee, So Yeon Kim, Manu Shivakumar, Kyu Joo Park, Young Jun Chai and Dokyoon Kim

Table S1. Selected features by Lasso-Cox feature selection.

Clinical features	Expression features	Methylation features	Expression+Methylation features
Age	CTNND1	CEP250	CEP250 (methylation)
N stage	DEFA5	GSDMA	DEFA5 (expression)
T stage	LRBA	IL18	MAZ (methylation)
Gender	MAPKAPK2	KIAA1109	NINJ1 (methylation)
	NLRP14	LPCAT3	NLRP14 (expression)
	NRAS	MAZ	PPARGC1A (expression)
	POU2F1	NINJ1	PRG4 (expression)
	PPARGC1A	RAB21	PTGES (expression)
	PRG4	SECISBP2	RAB21 (methylation)
	PRKDC	TNFRSF18	TERF2IP (expression)
	PTGES	TNFSF12	TMEM184A (expression)
	PTPN6	TNPO3	TNFRSF18 (methylation)
	RAB21		TNFSF12 (methylation)
	TERF2IP		TNPO3 (methylation)
	TMEM184A		
	TRAF5		
